# Dl‐3n‐butylphthalide improves traumatic brain injury recovery via inhibiting autophagy‐induced blood‐brain barrier disruption and cell apoptosis

**DOI:** 10.1111/jcmm.14691

**Published:** 2019-12-16

**Authors:** Fangfang Wu, Ke Xu, Kebin Xu, Chenhuai Teng, Man Zhang, Leilei Xia, Kairui Zhang, Lei Liu, Zaifeng Chen, Jian Xiao, Yanqing Wu, Hongyu Zhang, Daqing Chen

**Affiliations:** ^1^ Department of Emergency The Second Affiliated Hospital and Yuying Children's Hospital Wenzhou Medical University Wenzhou China; ^2^ Key Laboratory of Biotechnology and Pharmaceutical Engineering, School of Pharmaceutical Sciences Wenzhou Medical University Wenzhou China; ^3^ The Institute of Life Sciences, Engineering Laboratory of Zhejiang province for pharmaceutical development of growth factors,Biomedical Collaborative Innovation Center of Wenzhou Wenzhou University Wenzhou China; ^4^ Department of Pharmacy HwaMei Hospital, University of Chinese Academy of Sciences Ningbo China; ^5^ Department of Emergency Wenzhou People's Hospital, The Third Clinical Institute Affiliated to Wenzhou Medical University, Wenzhou Medical University Wenzhou China; ^6^ Department of Neurosurgery Affiliated Cixi Hospital, Wenzhou Medical University Ningbo China; ^7^ Experimental Research Centre Dongyang People's Hospital Wenzhou Medical University Jinhua China

**Keywords:** autophagy, blood‐brain barrier, Dl‐3n‐butylphthalide, mitochondrial apoptosis, traumatic brain injury

## Abstract

Blood‐brain barrier (BBB) disruption and neuronal apoptosis are important pathophysiological processes after traumatic brain injury (TBI). In clinical stroke, Dl‐3n‐butylphthalide (Dl‐NBP) has a neuroprotective effect with anti‐inflammatory, anti‐oxidative, anti‐apoptotic and mitochondrion‐protective functions. However, the effect and molecular mechanism of Dl‐NBP for TBI need to be further investigated. Here, we had used an animal model of TBI and SH‐SY5Y/human brain microvascular endothelial cells to explore it. We found that Dl‐NBP administration exerts a neuroprotective effect in TBI/OGD and BBB disorder, which up‐regulates the expression of tight junction proteins and promotes neuronal survival via inhibiting mitochondrial apoptosis. The expressions of autophagy‐related proteins, including ATG7, Beclin1 and LC3II, were significantly increased after TBI/OGD, and which were reversed by Dl‐NBP treatment both in vivo and in vitro. Moreover, rapamycin treatment had abolished the effect of Dl‐NBP for TBI recovery. Collectively, our current studies indicate that Dl‐NBP treatment improved locomotor functional recovery after TBI by inhibiting the activation of autophagy and consequently blocking the junction protein loss and neuronal apoptosis. Dl‐NBP, as an anti‐inflammatory and anti‐oxidative drug, may act as an effective strategy for TBI recovery.

## 1 INTRODUCTION

Traumatic brain injury (TBI) is one of the main causes of traumatic death and disability worldwide.^1^ The pathological mechanisms of TBI are divided into primary traumatic injury and secondary traumatic injury.^2^ Primary injuries lead to brain tissue disorganization, whereas secondary injuries result in cerebral oedema, intracranial hypertension, energy metabolism disorders, inflammation and extensive neuronal death.^3^, ^4^, ^5^, ^6^ The destruction of blood‐brain barrier (BBB) integrity is an important trigger of these complex cascades of molecular events.^4^ Therefore, reasonable BBB intervention in early stage of TBI would be an effective treatment strategy.^7^ Many methods have been attempted to repair the BBB after TBI, but there are limited successes.^8^ Another important pathological response after TBI is neuronal ‘programmed cell death’, which leads to lifelong disability.^9^ Inhibition of neuronal apoptosis after TBI may be an effective initial treatment.^10^, ^11^, ^12^ Thus, the drugs that reduce BBB disorders and alleviate apoptosis may decrease clinical complications and improve the prognosis of TBI.^2^

Autophagy relies on the lysosomal pathway to degrade damaged cytoplasmic proteins and ageing organelles, and thereby recycle the cell structure.^13^ Autophagy plays a cellular protective role under stress, cell ageing and cell inflammation condition. In addition to its role in cellular homeostasis, autophagy also contributes to programmed cell death,^14^ which can induced apoptosis via enhancing tumour necrosis factor‐related apoptosis‐inducing ligand (TRAIL).^15^ A growing body of evidences indicate that autophagy is important for the functional recovery of several central nervous system (CNS) diseases.^16^, ^17^, ^18^ Inhibition of autophagy activation is beneficial for BBB integrity in early stage in stroke. Enhanced autophagy by rapamycin (RAPA) further causes ZO‐1 degradation and MMP‐9 secretion after high glucose‐induced damage.^19^ An increasing number of studies have demonstrated that autophagy may be involved in the pathophysiological process of TBI.^6^, ^10^, ^20^ Many damaged neurons exhibit the characteristics of autophagic/lysosome cell death post‐TBI, such as induction of GFP‐LC3 immunofluorescence and accumulation of the autophagic substrate P62.^21^ In neonatal brain injury, Atg7‐deficient mice show less neuronal death, leading to reduced tissue loss.^22^ However, the role of autophagy after TBI, especially BBB disruption and apoptosis, and the development of a new approach to reverse their damaging effects need to be further study.^23^

Dl‐3n‐butylphthalide (Dl‐NBP),a chemical compound, is originally extracted from the seeds of Chinese celery, *Apium graveolens Lin*.^24^ Multiple studies have demonstrated that Dl‐NBP exerts a neuroprotective effect in cerebral ischaemia, probably due to its ability to decrease brain oedema, and thereby suppress oxidative stress, reduce neuronal death and promote anti‐inflammation.^24^ Additionally, our previous work suggested that Dl‐NBP ameliorates locomotor functional recovery in animal model of spinal cord injury (SCI)^25^ and L‐NBP enhances cognitive function in Alzheimer's disease.^26^ Furthermore, L‐NBP inhibits autophagy and improves cognitive impairment in mice with repeated cerebral ischaemia‐reperfusion injury.^27^ Recently, it is reported that Dl‐NBP promotes regenerative repair and functional recovery of mice after TBI.^28^ However, the mechanisms underlying the protective effect of Dl‐NBP in acute TBI has not previously been investigated.

In this study, a TBI mouse model created by controlled cortical impact (CCI) was used to assess the beneficial effect of Dl‐NBP on TBI recovery. During mechanism study, we have found that autophagy is involved in the neuroprotective function of Dl‐NBP after TBI, which blocks the junction protein loss and neuron apoptosis. Our current study suggests that Dl‐NBP, as an anti‐inflammatory and anti‐oxidative drug, holds a great promise to develop a new treatment for TBI recovery.

## 2 MATERIALS AND METHODS

### 2.1 Animals and surgical procedures

Adult C57BL/6 male mice aged 6‐8 weeks and weighing 20‐26 g were purchased from the Animal Center of the Chinese Academy of Science (Shanghai, China). Animals were housed under a 12‐hour light/dark cycle at 21‐23°C and provided access to food and water ad libitum. All experimental procedures were approved by the Ethics Committee of Wenzhou Medical University and conformed to the Guide for the Care and Use of Laboratory Animals from the National Institutes of Health. The experiments were performed according to the graphical timeline in Figure S1A. For TBI induction, male mice were anaesthetized with isoflurane in the brain stereotaxic apparatus. For each mouse, the head fur was shaved and sterilized with iodophors, and an incision was made in the middle of the scalp to expose the skull. To expose the cortex, a hand‐held dental drill was used to remove a bone flap (5 mm from the upper left temporal lobe), while keeping the dura intact. Controlled cortical impact was performed by a controlled impactor device with a speed of 4 m/s, a depth of 1 mm and a 150‐ms impact duration (Impact One™Stereotaxic Impactor, Leica). The sham‐operated group received only anaesthesia and a craniotomy. The incision was sutured, and the local antibiotic (cefazolin sodium salt) was applied to the incision site. Mice were placed on a heating pad at 37°C to recover from anaesthesia. In the TBI + Dl‐NBP group, Dl‐NBP was diluted in a mixture of castor oil and PBS (1:15). The drug was filtered by 0.2 μm filtration before injection. The daily injection dose was 100 mg/kg. Every mouse was injected with about 200 μL working solution, which was determined by mice weight. After injury, Dl‐NBP was administered by intracerebroventricularly injection and then by oral gavage once daily for one week until sacrifice. RAPA, an autophagy inducer, was injected intraperitoneally (0.5 mg/kg) in the TBI + Dl‐NBP + RAPA group. 3‐MA, a selective PI3K inhibitor, was administered in a similar fashion via intraperitoneal injection (15 mg/kg) in the TBI + 3‐MA group. Regarding the method of drug administration, RAPA was injected 1 d after Dl‐NBP treatment, and then, RAPA/3‐MA and Dl‐NBP were simultaneously administered once daily for one week. Vehicle control mice were injected with a sham volume of solvent. All experiments were conducted with all efforts to reduce the number of animals used and suffering as much as possible.

### 2.2 Behavioural test

The Garcia test was conducted by researchers unaware of the experimental conditions at 1, 3 and 7 d after CCI impact. The Garcia test score is a 21‐point scale (score 0‐21) that includes seven items, namely spontaneous activity, axial sensation, vibrissae proprioception, limb symmetry, lateral turning, forelimb outstretching and climbing. The score of each item ranged from 0 (worst performance) to 3 (best performance), with the sum of each item totalling the Garcia test score.

### 2.3 Evans Blue (EB) extravasation assay

To examine BBB integrity, EB extravasation assays were performed. After TBI, mice were injected with 0.25 mL EB dye (2%) via the caudal vein. Two hours later, the animals were anaesthetized and killed by saline infusion. The left hemisphere of each brain was weighed, added into 500 µL N,N‐dimethylformamide and incubated at 72°C for 3 d. Samples were centrifuged twice at 1516 *g* for 20 minutes. The supernatant was collected and aliquoted (200 μL) into a 96‐well glass plate. Fluorescence was quantified using a spectrophotometer at an excitation wavelength of 620 nm and an emission wavelength of 680 nm. All experiments were repeated at least in triplicate.

### 2.4 Ultrastructural observation of nerve cells

Neuronal ultrastructural morphology was observed by transmission electron microscopy (TEM). Brain tissues were cut into 1‐mm sections, fixed overnight with 2.5% glutaraldehyde, post‐fixed in 2% osmium tetroxide and blocked with 2% uranylacetate. After dehydration in acetone, the tissue was placed in Araldite, and semi‐thin sections were stained with toluidine blue to determine the ultrastructure. At least six slices were observed for each sample, and a minimum of 30 fields of vision were analysed.

### 2.5 Cell culture and OGD/re‐oxygenation model

SH‐SY5Y cells were cultured in Dulbecco's modified Eagle's medium (DMEM, Invitrogen) supplemented with 10% foetal bovine serum (FBS, Gibco), 100 U/mL penicillin and 100 µg/mL streptomycin (Gibco). Primary cultured HBMECs were purchased from ScienCell Research Laboratories. HBMECs were cultured in Endothelial Cell Medium (ECM, ScienCell) supplemented with 5% FBS (ScienCell), 1% ECGS (ScienCell) and antibiotics (100 U/mL penicillin and 100 µg/mL streptomycin, ScienCell). Cells were then incubated in a humidified atmosphere containing 5% CO2 at 37°C. NBP was diluted to a stock solution of 10 mmol/L in DMSO. Cells were treated with OGD. For OGD, normal growth medium was replaced with FBS‐free ECM, and cells were incubated in an anaerobic chamber for 6 hours in which the oxygen level remained below 0.5%. After OGD, cells continued to incubate for 12 hours under normal culture conditions. NBP (10 μmol/L) pre‐treatment lasted for 2 hours before OGD and continued during the re‐oxygenation process. To further estimate the effect of autophagy activation on OGD, cells were pre‐treated with RAPA (100 nmol/L) and 3‐MA (5 μmol/L) for 1 hour.

### 2.6 Western blot analysis

Animal tissues and cells were lysed with RIPA buffer (pH 7.4, 50 mmol/L Tris, 150 mmol/L NaCl, 1% Triton X‐100, 1% sodium deoxycholate, 0.1% SDS, sodium orthovanadate, sodium fluoride and EDTA). Tissue homogenates were centrifuged at 12 000 rpm, for 15 minutes at 4°C. The extracted supernatant was quantified by the BCA assay (Thermo). Total protein (40 μg) was separated on a 12% gel and transferred onto a PVDF membrane (Bio‐Rad). The membranes were blocked for 1.5 hours in 5% dry milk dissolved in 0.1% Tween‐20 in TBS at room temperature and incubated overnight with the following primary antibodies: P120‐catenin (1:1000, Abcam), β‐catenin (1:1000, Abcam), occludin (1:1000, Abcam), cleaved caspase‐3 (1:1000, Abcam), Bcl‐2 (1:1000, Abcam), Bax (1:1000, Abcam), Tomm20 (1:1000, Abcam), ATG7(1:1000, Novus), beclin1 (1:1000, Abcam), LC3II (1:1000, Novus) and GAPDH (1:10 000, Yeasen). Then, the membrane was washed three times with TBST and incubated with a horseradish peroxidase conjugated secondary antibody. A ChemiDoc™ XRS imaging system (Bio‐Rad) was used to visualize the signals. Quantity one was used to analyse the relative density of the bands, and band density was normalized to that of GAPDH. All experiments were repeated at least in triplicate.

### 2.7 TUNEL staining

The terminal deoxynucleotidyl transferase (TdT)‐mediated dUTP nick end labelling (TUNEL) staining was used to test apoptosis level according to the manufacturer's protocol (YEASEN, 40307ES60). Briefly, after dewaxing and hydration, the brain sections were incubated with 20 μg/mL proteinase K working solution for 15 minutes at 37°C. The slides were then rinsed with PBS for three times, which was followed by incubation with the TUNEL reaction mixture for 1 hours at 37°C. After rinsing with PBS for three times, the sections were stained with 4′, 6‐diamidino‐2‐phenylindole (DAPI, Beyotime) for 5 minutes at room temperature and mounted with aqueous mounting medium. All images were captured using a laser scanning confocal microscope. TUNEL‐positive cells were calculated by using ipwin software.

### 2.8 Immunofluorescence staining

Brain tissue was embedded in paraffin, cut into 5‐mm sections and mounted onto slides. Tissues were deparaffinized. Cells were fixed with 4% paraformaldehyde (PFA) for 30 minutes at 37°C. Tissues and cells were blocked in 5% bovine serum albumin (BSA) for 30 minutes and then incubated at 4°C overnight with the following primary antibodies: Claudin‐5 (1:500, Abcam), cleaved caspase‐3 (1:500, Abcam), LC3II (1:500, Novus) and NeuN (1:500, Abcam).The next day, sections and cells were incubated with the corresponding secondary antibody: Alexa Fluor594/647 donkey antimouse/rabbit antibody (1:1000, Abcam) for 1 hour at 37°C. Nuclei were stained with DAPI, and cells were imaged under a Nikon ECLIPSE Ti microscope (Nikon, A1 PLUS).

### 2.9 Statistical analysis

Statistically analysed data are presented as the mean ± SEM. Student's *t* test was used to determine statistical significance between two groups. For comparison of groups of three or more, one‐way analysis of variance (ANOVA) followed by Tukey's post hoc test was used to analyse the results. The difference was considered statistically significant when the *P* value was <.05.

## 3 RESULTS

### 3.1 Dl‐NBP treatment improves mouse motor function via restoring BBB permeability and reducing neuronal cell death after TBI

**Figure 1:**
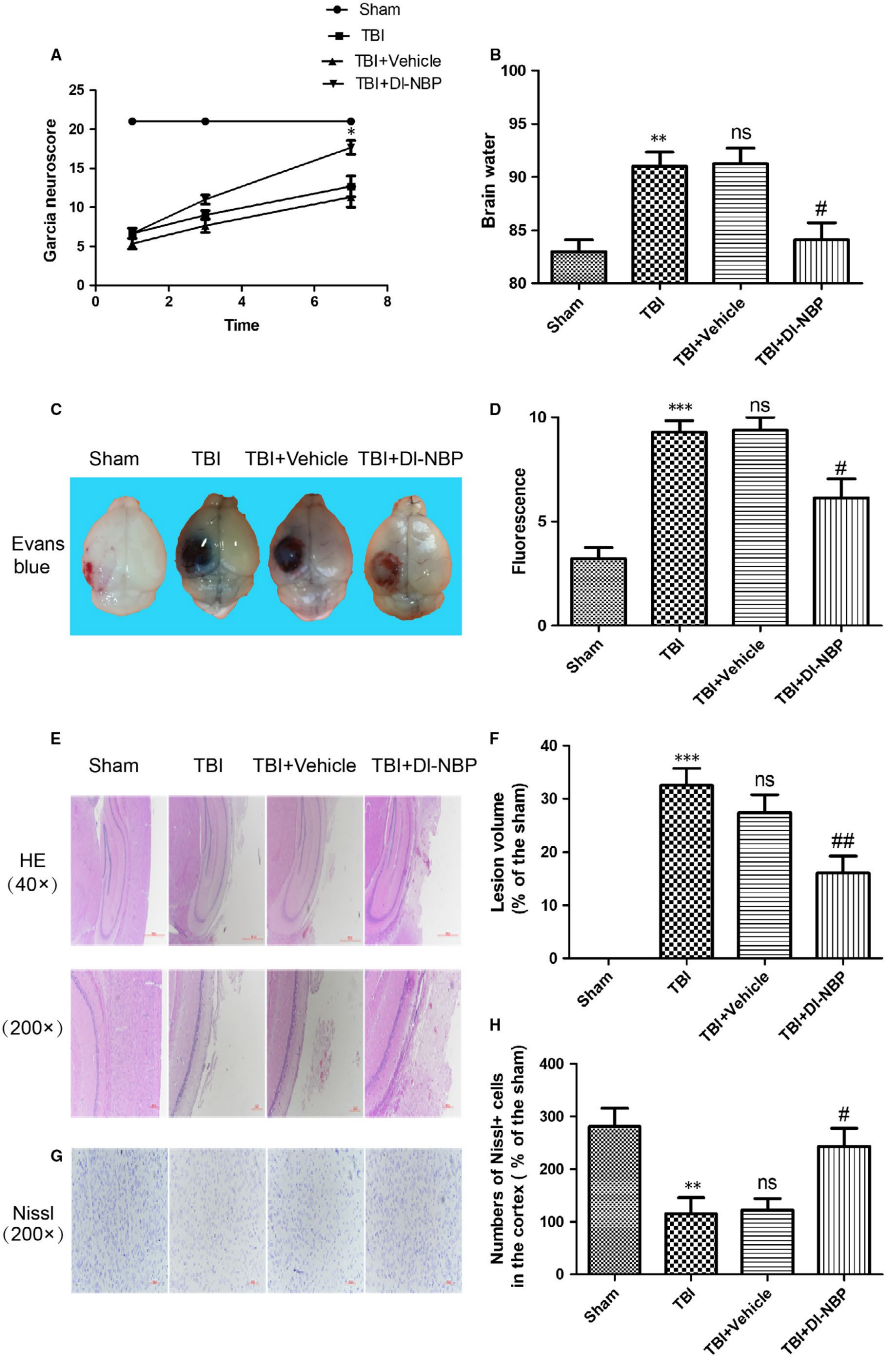
Dl‐NBP treatment improves functional recovery after TBI. A, Garcia test evaluation at 1, 3 and 7 d after TBI. B, Quantification of brain water content in the ipsilateral brain cortex at 3 d after TBI. C, Representative images of the brains with Evans Blue dye staining at 3 d post‐TBI. D, Quantification of the EB dye leakage in the brain of mice. E, Representative images of haematoxylin and eosin (H&E) staining in the cortex at 7 d post‐TBI. F, Quantification of lesion volume after H&E staining; G, Representative images of Nissl staining in the cortex at 7 d post‐TBI. H, Quantification analysis of Nissl staining. Data represent the mean value ± SEM, n = 6. **P < .01 and ***P < .001 vs the sham group, ns and #*P* < .05 vs the TBI group

To evaluate the effect of Dl‐NBP on TBI, the mice were intracerebroventricularly injected with Dl‐NBP at day 1 and subsequently received Dl‐NBP via oral gavage. As shown in Figure 1A, the Garcia scores were not significantly different between the Dl‐NBP‐treated and TBI group or between the TBI and TBI + Vehicle group at days 1 and 3. Interestingly, there was significantly increased in the Dl‐NBP‐treated group compared with those in TBI and TBI + Vehicle groups at day 7 during 21‐point Garcia test (*P* < .05; Figure 1A). To estimate cerebral oedema, brain water content was evaluated at day 7 after TBI. Brain water content was significantly increased in TBI group when compared with that in the sham group (91.03 ± 1.323 vs 83.01 ± 1.117, respectively; Figure 1B). The brain water content did not differ significantly between TBI and TBI + Vehicle group (91.03 ± 1.323 vs 91.28 ± 1.455). Compared with TBI group, the Dl‐NBP‐treated group showed a substantial reduction in the percentage of brain water content (84.11 ± 1.605 vs 91.03 ± 1.323, respectively; Figure 1B). Then, we examined BBB permeability at day 3 after TBI via the Evans Blue (EB) assay. The BBB permeability was increased in the TBI and TBI + Vehicle group compared with that in the control group. However, the Dl‐NBP‐treated group had significantly reduced EB intake (Figure 1C,1D) compared with the injured group.

Next, we used H&E staining and Nissl staining to assess histological morphology in each group. As shown in Figure 1E,1F, severe cerebral cortex tissue loss was observed in the TBI and TBI + Vehicle group, which was reversed in the Dl‐NBP‐treated group. The number of neuron in cerebral cortex of mice was significantly reduced in the TBI and TBI + Vehicle groups when compared with that the sham group, which was significantly blocked by Dl‐NBP treatment (Figure 1G,H, *P* < .05). In summary, these results indicate that Dl‐NBP ameliorates motor dysfunction by alleviating BBB disruption and reducing neuronal necrosis, indicating that Dl‐NBP has a protective effect on TBI recovery.

### 3.2 Dl‐NBP preserves the reduction of tight junctions and adherens junctions

**Figure 2:**
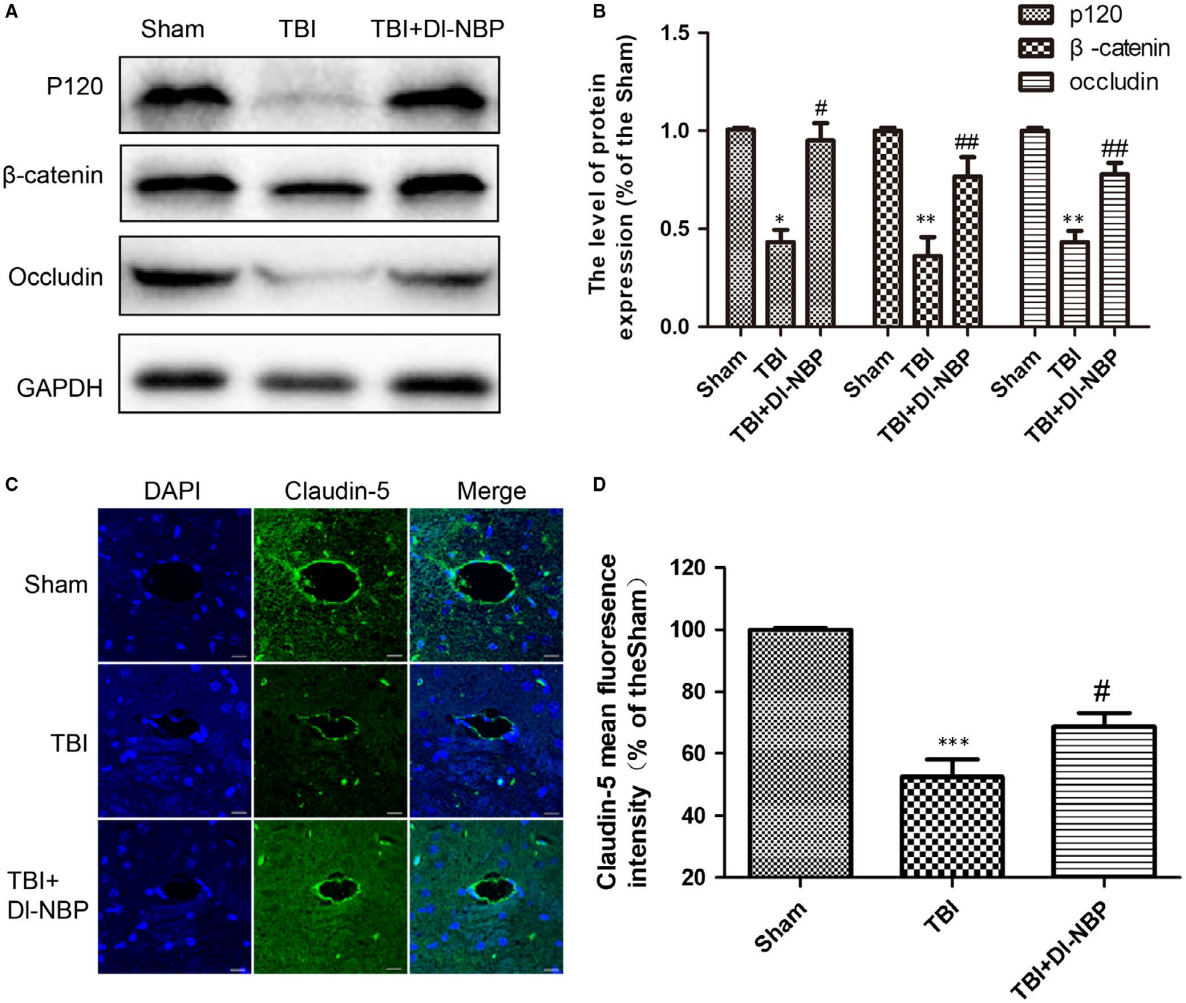
Dl‐NBP prevents BBB disruption by improving the expressions of junction proteins at 3 d post‐TBI. A, Representative Western blot analyses of P120‐catenin, β‐catenin and occludin in the cerebral cortex. B, Quantification of the Western blot data for the junction proteins. C, Representative fluorescence images of claudin‐5(green) and DAPI stained nuclei(blue). Scale bar = 100 μm. D, Quantification of fluorescence intensity from (C). Data represent the mean value ± SEM, n = 6. **P < .01 and ***P < .001 vs the sham group; #*P* < .05 and ##*P* < .01 vs the TBI group

To further determine whether Dl‐NBP exerts its neuroprotective effect by repairing BBB damage after TBI, we evaluated expression levels of tight junction (TJ) proteins and adherens junction (AJ) proteins using Western blotting. TJ (occludin) and AJ (P120‐catenin and β‐catenin) protein expression in the cortex was markedly reduced in the TBI group compared with those in the sham‐operated group at day 3 post‐injury. To a certain extent, Dl‐NBP treatment reversed it (Figure 2A,2B). As shown in Figure 2C,2D, the immunofluorescence staining also revealed that the optical density of claudin‐5 in the cortical lesions was substantially decreased in TBI group when compared with that in the sham group, which was reversed by Dl‐NBP treatment. These data suggest that Dl‐NBP prevents the destruction of BBB integrity after TBI.

### 3.3 Dl‐NBP reduces neuronal apoptosis by protecting mitochondrial function

**Figure 3:**
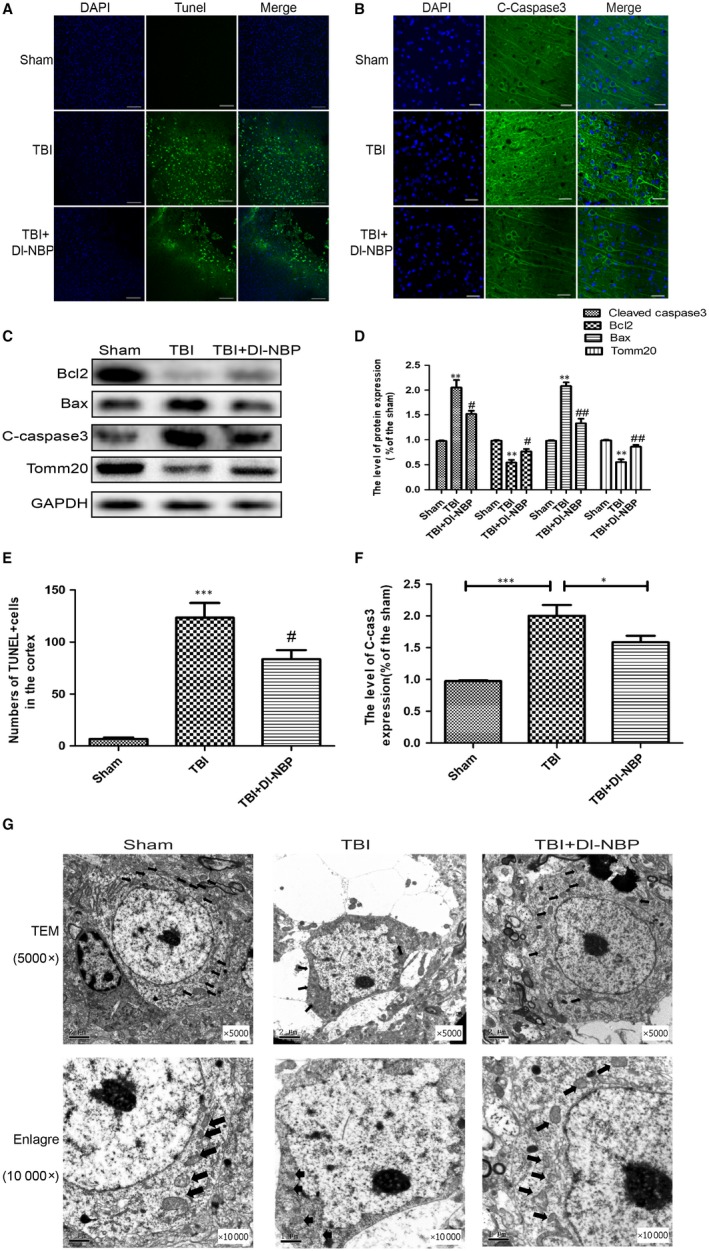
Dl‐NBP protects against neuronal apoptosis at 3 d post‐TBI. A, Immunofluorescence staining of TUNEL (green) in the cortex at 3 d post‐injury. Scale bar = 100 μm. B, Immunofluorescence staining of cleaved caspase‐3(green) in the cortex at 3 d post‐injury. Scale bar = 20 μm. C, Representative Western blot of the expression of apoptosis‐related proteins in the cortex in the different groups at 3 d after TBI. D, E, F, Quantification analysis from (A, B and C). G, Transmission electron microscopy (TEM) of brain lesions in the different groups at 3 d after TBI (magnification ×5000 and ×10 000). Data represent the mean value ± SEM. ***P < .001, **P < .01 and *P < .05 vs the sham group; #*P* < .05 and ##*P* < .01 vs the TBI group; n = 6 per group

Then, we tested the impact of Dl‐NBP on neuronal apoptosis (cleaved caspase‐3) and the expressions of mitochondrial apoptosis‐related proteins (Bcl‐2 and Bax) in the cortex at day 3 post‐TBI. As shown in Figure 3A,3B, the number of TUNEL‐positive (green) and cleaved caspase‐3‐positive (green) cells in the cortex was substantially increased at day 3 after TBI. Dl‐NBP treatment decreased the number of apoptotic cells. Moreover, as shown in Figure 3C‐F, Dl‐NBP treatment decreased neuron apoptosis post‐TBI and down‐regulated the expressions of pro‐apoptotic protein. We also detected the expression of Tomm20, which acts as a transit peptide receptor at the surface of the mitochondrial outer membrane. As shown in Figure 3C, Dl‐NBP reversed TBI‐induced suppression of Tomm20 expression. These data suggest that Dl‐NBP inhibits mitochondrial apoptosis signalling in the cortex of brain after TBI.

Then, we used transmission electron microscopy to further examine the ultrastructure of neuron (Figure 3G). In the sham group, the neuron exists normal morphology with the dendrite, axon and cell body, which has integrated cell membrane large and round nuclei, shollow chromatin and normal mitochondria. However, the TBI group had a large amount of cell debris, which had unclear outlines, ruptured cell membrane, pyknotic nuclei and disintegrated organelles (especially mitochondria) in the cytoplasm. These phenomenons were reversed by Dl‐NBP treatment. Taken together, these data suggest that Dl‐NBP administration greatly alleviates TBI‐induced neuron apoptosis, and this effect is related to mitochondria.

### 3.4 Dl‐NBP down‐regulates the expressions of autophagy‐related proteins

**Figure 4:**
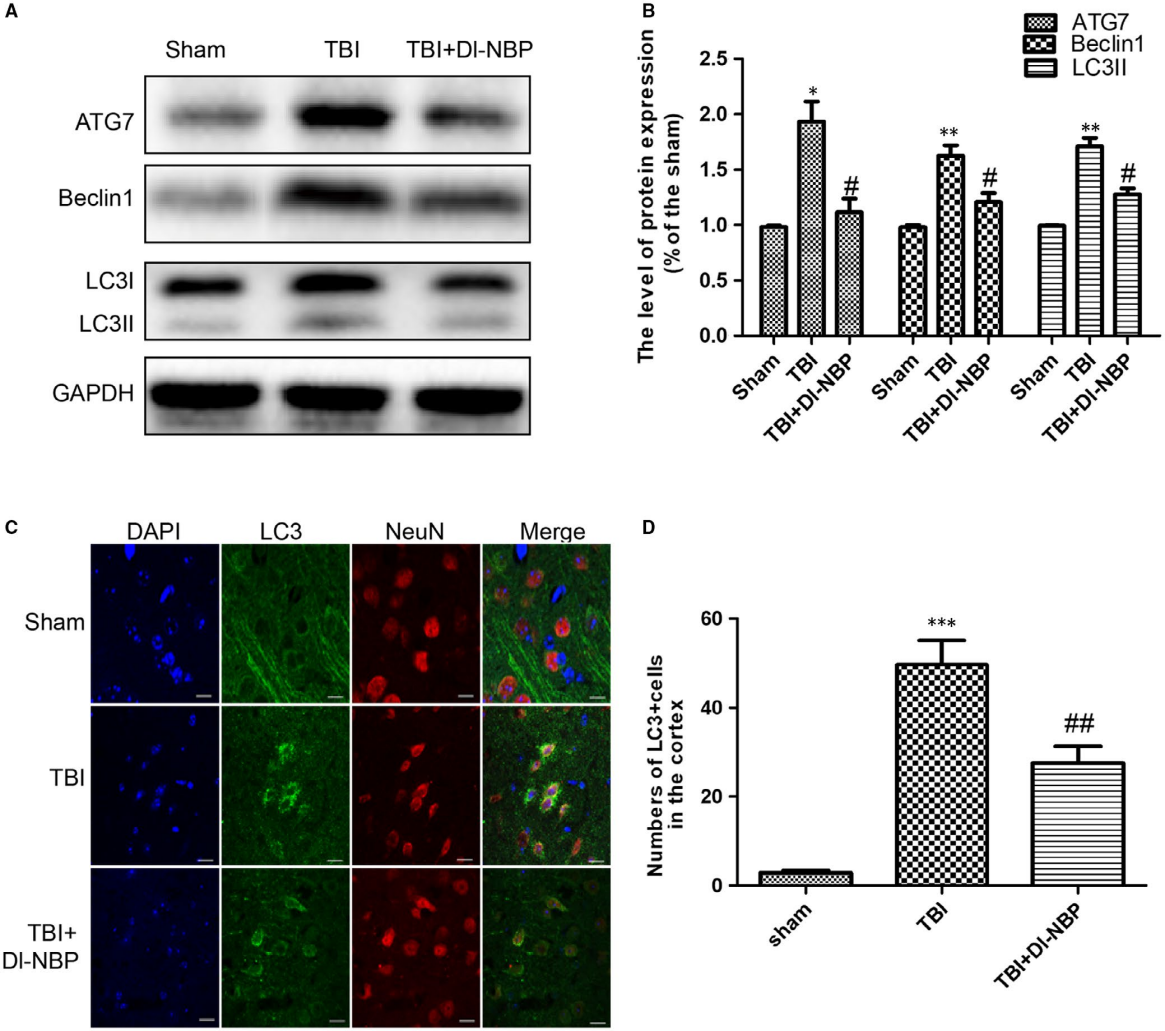
Dl‐NBP treatment inhibits TBI‐induced autophagy activation at 3 d post‐injury. A, Representative Western blots of the expressions of ATG7, beclin1 and LC3B in the cerebral cortex 3 d post‐injury. B, Western blot analysis of the data in (A). C, Representative fluorescent images depicting LC3 (green) with NeuN (red) in the cortex after TBI. Scale bar = 10 µm; n = 6. D, Quantitative analysis of LC3 fluorescent results. All data represent the mean value ± SEM, n = 6. ***P < .001 and **P < .01 vs the sham group; #*P* < .05 and ##*P* < .01 vs the TBI group

Recent studies have shown that the autophagy level is significantly increased following TBI.^29^ To further investigate whether Dl‐NBP reduces autophagy after TBI, we examined the expressions of autophagy‐related proteins (ATG7, Beclin1 and LC3II) using Western blotting. As shown in Figure 4A,4B, Dl‐NBP treatment attenuated CCI‐induced autophagy. To further examine neuronal autophagy, we performed double staining of NeuN and LC3II (Figure 4C,4D). Consistent with Western blotting, the number of LC3II‐positive neurons in the Dl‐NBP‐treated group was significantly decreased. These findings demonstrate that Dl‐NBP inhibits autophagy activation after TBI.

### 3.5 RAPA treatment abolishes the effect of Dl‐NBP on maintaining of BBB integrity

**Figure 5:**
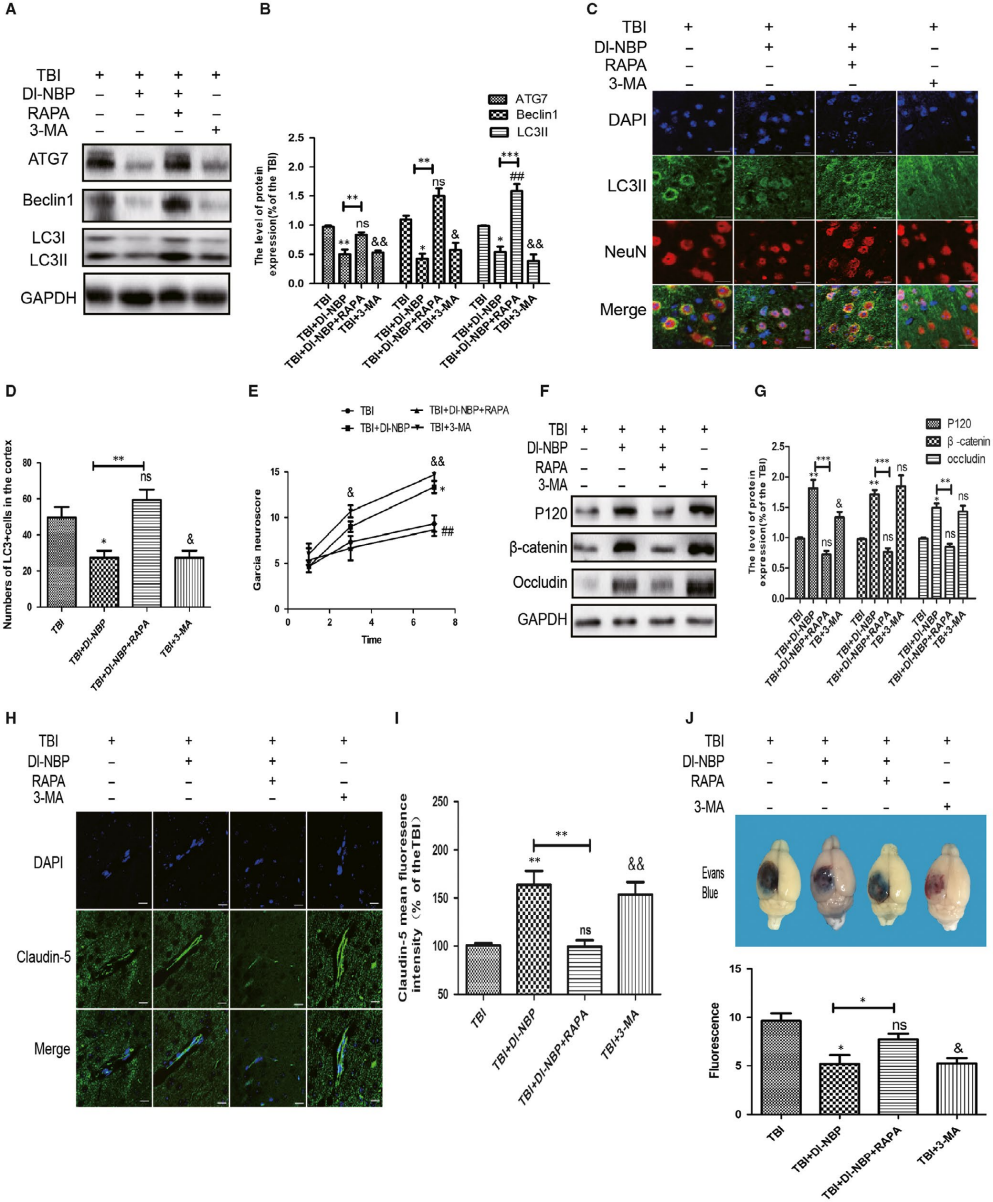
RAPA treatment abolishes the effect of Dl‐NBP on maintaining of BBB integrity. A, Representative Western blots showing the expression of autophagy‐related proteins in the cerebral cortex. B, Quantification of the Western blot data from (A). C, Representative fluorescent images depicting LC3 (green) with NeuN (red) in the cortex after TBI. Scale bar = 10 µm; n = 6. D, Quantitative analysis of LC3 fluorescent results. All data represent the mean value ± SEM, n = 6. *P < .05 and ns vs the TBI group; #*P* < .05 vs the TBI group. **P < .01 vs the indicated group; E, Garcia test evaluation at 1, 3, 7 d between the group. Data are the mean value ± SEM, n = 3, *P < .05, &*P* < .05 and &&*P* < .01 vs the TBI group. *P < .05 vs the TBI + NBP group. F, G, Representative Western blot and quantification data of junction protein expressions in each group. H, I, Immunofluorescence staining and quantification of claudin‐5 (green) in the cortex at 3 d post‐injury. Scale bar = 100 µm. All data represent the mean value ± SEM, and n = 6 in all *t* tests. **P < .01, ns, &&*P* < .01 vs the TBI group; **P < .01 vs the indicated group. J, Representative and quantification of Evan's Blue dye permeability data at 3 d post‐TBI. *P < .05 vs the TBI group; ns vs the TBI group; &*P* < .05 vs the TBI group; n = 6

To investigate the relationship between autophagy and Dl‐NBP neuroprotective role, we used rapamycin and 3‐MA (an autophagy inhibitor) to treat the mice after TBI. As shown in Figure 5A‐D, the expressions of autophagy‐related proteins were substantially higher in the Dl‐NBP and RAPA co‐treated group than those in Dl‐NBP‐treated group at day 3 post‐TBI, indicating that RAPA reverses the effect of Dl‐NBP on autophagy. As shown in Figure 5E, the Garcia scores were not significantly different between the TBI + Dl‐NBP and TBI + Dl‐NBP + RAPA group at day 1 and day 3. However, the Garcia scores were significantly increased at day 7 in the Dl‐NBP‐treated group compared with that in the co‐treated group (##*P* < .01). Then, we detected TJ and AJ protein expression by Western blotting and immunofluorescence staining. As expected, TJ and AJ protein levels were increased in the Dl‐NBP treatment group, whereas those levels were decreased in the Dl‐NBP + RAPA treatment group (Figure 5F‐I) when compared with that in Dl‐NBP treatment group. As shown in Figure 5J, compared with the Dl‐NBP‐treated group, the EB content was significantly increased in the co‐treated group. These data demonstrate that suppressing autophagy contributes to the neuroprotective effect of Dl‐NBP, which is similar to the effect of 3‐MA.

### 3.6 The beneficial effect of Dl‐NBP on apoptosis is suppressed by RAPA treatment

**Figure 6:**
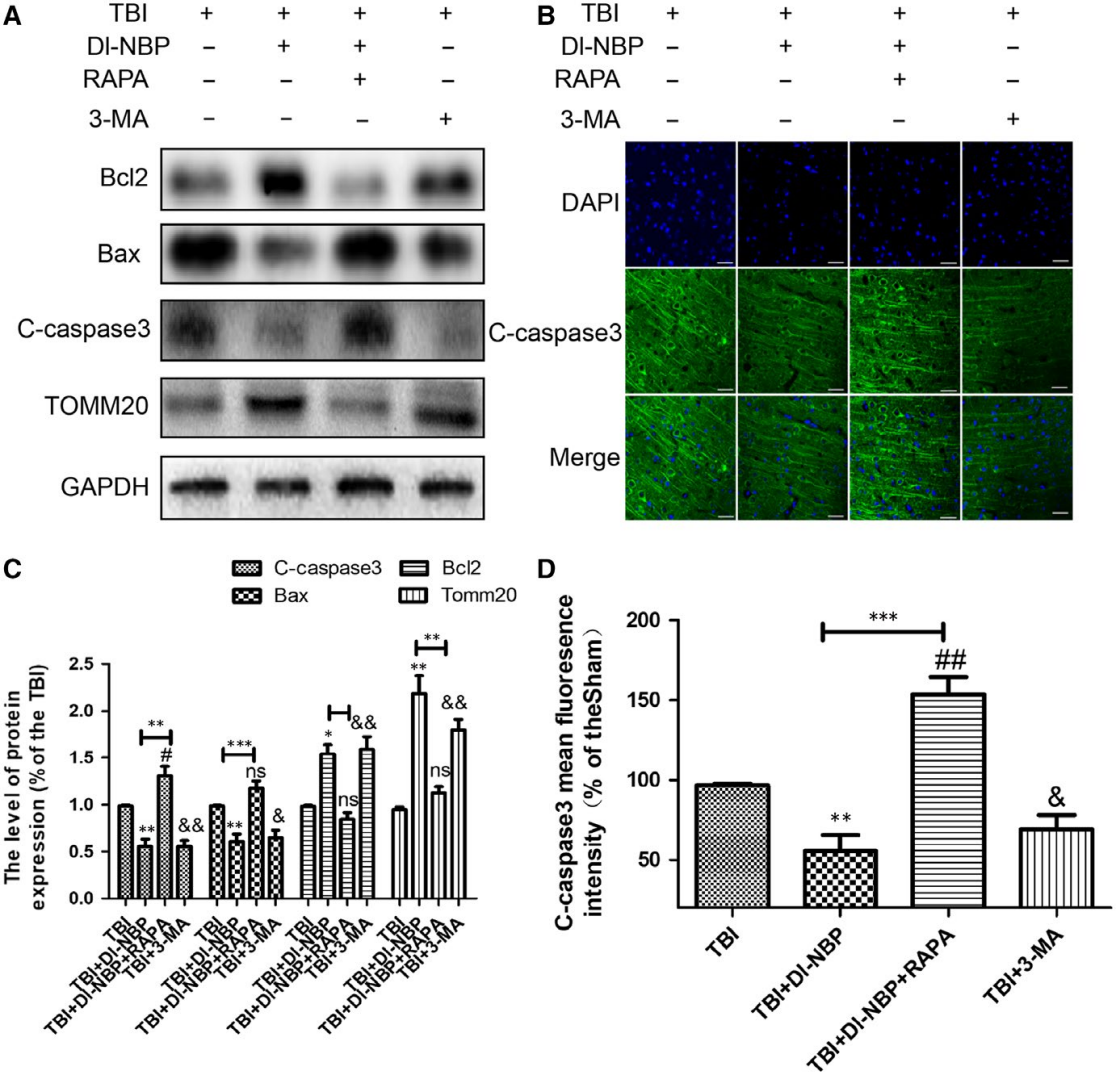
The beneficial effect of Dl‐NBP on cell apoptosis was suppressed by RAPA treatment. A, Representative Western blots showing the expressions of apoptosis‐related proteins and Tomm20 protein after injury. B, Immunofluorescence staining of cleaved caspase‐3 in the cortex. Scale bar = 20 μm. C, D, Quantification of the Western blot and immunofluorescence staining data from (A and B). All data represent the mean value ± SEM, n = 6. *P < .05, **P < .01 and ***P < .001 vs the TBI group; ns, #*P* < .05, and ##*P* < 0.01 vs the TBI group; &*P* < .05, &&*P* < .01 and &&&*P* < .001 vs the TBI group;**P < .01 and ***P < .001 vs the indicated group

Next, we measured the role of autophagy during Dl‐NBP regulating mitochondrial apoptosis‐related protein levels at day 3 after TBI. The expressions of pro‐apoptotic proteins (cleaved caspase‐3 and Bax) were higher in the NBP + RAPA co‐treatment group than those in the Dl‐NBP‐treated group (Figure 6A‐D). NBP + RAPA co‐treatment decreased Tomm20 expression when compared with that in the Dl‐NBP‐treated group (Figure 6A,6C). These data indicate that RAPA, a specific inducer of autophagy, eliminates the protective effect of Dl‐NBP on mitochondrial apoptosis after TBI.

### 3.7 RAPA treatment abolishes the protective effect of Dl‐NBP on BBB integrity and cell survival in vitro

To further confirm the effect of Dl‐NBP on BBB integrity and neuronal survival, we detected mitochondrial apoptosis levels in SH‐SY5Y cells after OGD. As shown in Figure S2A,C, Dl‐NBP inhibited mitochondrial apoptosis after OGD. Then, we evaluated the effect of Dl‐NBP on autophagy by Western blotting. Dl‐NBP ameliorated OGD‐induced autophagy in SH‐SY5Y cells (Figure S2B,D). Moreover, AJ and TJ protein levels in HBMECs were increased in Dl‐NBP‐treated cells and decreased in Dl‐NBP + RAPA co‐treated cells (Figure S2E,G,H). Additionally, mitochondrial activity was higher in the DL‐NBP group than that in the OGD group (Figure S2F). Taken together, these results reveal that Dl‐NBP treatment inhibits the mitochondrial apoptosis and reduces the destruction of BBB integrity in vitro.

## 4 DISCUSSION

Although advances in science and evidence‐based medicine, the mortality and disability of patients with severe TBI has is still high.^30^ Thus, it is urgent need to find a drug that effectively inhibits the pathological development of TBI in the early stage.^2^ In the present study, we treated TBI mice with Dl‐NBP and found that Dl‐NBP exerts its neuroprotective effect and promotes the recovery of TBI. Mechanism studies have further observed that the neuroprotective role of Dl‐NBP after TBI was correlated with the down‐regulation of autophagy and consequently ameliorated BBB disorder and neuronal apoptosis.

The BBB strictly restricts neurotoxic substances, inflammatory factors and immunological cells and excretes metabolites and neurotoxic substances from CNS, thereby maintaining the homeostasis of brain microenvironment and ensuring normal nervous system function.^31^ BBB breakdown occurs within hours or days after TBI accompanying with oedema, neuroinflammation and cell death.^32^ Our previous study showed that basic fibroblast growth factor (bFGF) inhibits BBB disorders through PI3K‐Akt‐Rac1 signalling and thus improves functional recovery after TBI.^33^ Dl‐NBP is widely used to treat for ischaemic stroke patients.^34^ There are study showing that Dl‐NBP treatment attenuates BBB disruption, reduces EB dye extravasation and decreases the need for immunoglobulin treatment in cerebral ischaemia‐reperfusion injury.^35^ Our previous study suggested that Dl‐NBP prevents blood‐spinal cord barrier disruption (BSCB) after SCI by inhibiting endoplasmic reticulum (ER) stress.^25^ In current study, we found that Dl‐NBP reduced EB leakage and protected against cerebral oedema by improving expressions of junction proteins after TBI.

Autophagy is a conservative method of cell self‐degradation, which allows the degradation and reutilization of damaged organelles and macromolecules through lysosomes.^13^ It has demonstrated that the autophagy activation is involved in the progression of TBI, indicating that an increase in beclin1 occurs at the early stage of injury (4 hours) in neurons, and at 3 days in astrocytes, lasting for at least 3 weeks in all cell types.^36^ Meanwhile, some studies have reported that Dl‐NBP inhibits autophagy and thus reduces β‐amyloid generation and secretase activity in neuroblastoma 2a/amyloid precursor protein 695 cells treated with OGD.^37^ Dl‐NBP induces translocation of Nrf2 from the cytoplasm to nucleus and increases expressions of its downstream proteins after TBI.^38^ Based on these studies, our study has firstly demonstrated that the neuroprotective effect of Dl‐NBP on TBI is associated with down‐regulation of autophagy with suppression of ATG7, beclin1 and LC3II both in vivo and in vitro.

The regulation of autophagy on cells is twofold: moderate autophagy promotes cell survival in harmful conditions to a certain degree, whereas severe or rapid autophagy induces programmed cell death, which is known as autophagic cell death.^39^ Autophagy and apoptosis are both strictly controlled biological processes that play important roles in development, tissue homeostasis and disease.^39^ There are complex interactions between autophagy and apoptosis. Beclin1 plays an important role in this network interaction. Beclin1, a gatekeeper involving in the formation of early autophagosomes, also participates in the regulation of mitochondrial apoptosis pathways.^40^

Traumatic brain injury causes activation of mitochondrial apoptosis.^17^, ^36^ Rats intravenously injected with Dl‐NBP on a daily basis has showed that NBP treatment also significantly reduced reactive astrogliosis and apoptosis, and protected hippocampal neurons against resistance ischaemic injury in a model of vascular dementia, which lasts for 14 days.^41^ Our previous studies have reported that rats treated with Dl‐NBP improved locomotor recovery after SCI by inhibiting ER stress‐induced apoptosis, which lasts 28 days.^25^ In our study, we found that Dl‐NBP administration ameliorated mitochondrial apoptosis and BBB damage in TBI/OGD, which consistent with the results of prior studies. In addition, our previous study showed that the down‐regulation of LC3II and enhanced clearance of ubiquitin protein by p62 were correlated with nerve survival and BSCB integrity following SCI.^42^ It has been suggested that the up‐regulation of beclin1 and decrease in lysosome‐associated membrane protein‐2 were accompanied by increased ROS, leading to cell death in re‐oxygenation injury.^43^ Interestingly, Zhou's study has also showed that the up‐regulation of autophagy has therapeutic effects in SCI, which reduces ER stress‐dependent apoptosis, thereby protecting against BBB disorders after SCI.^44^ However, the relationship among cellular stress, autophagy and apoptosis after TBI is unclear, which need to be further study. In our current study, Dl‐NBP down‐regulated autophagy and improved the functional recovery of TBI by reducing mitochondrial apoptosis and preserving BBB integrity by treating the mice with RAPA and 3‐MA. These results further confirm that autophagy is involved in the neuroprotective function of Dl‐NBP.

Previous studies have indicated that autophagy has an important function both in the TBI and SCI, and however, it is full of controversy the role of autophagy in TBI and SCI, especially in BSCB. It has been reported that lithium chloride enhances the BSCB after 1d of SCI by stimulating autophagy.^45^ Some studies have been also reported that inhibiting the TBI‐inducing autophagy alleviates BBB disruption.^46^, ^47^ These studies have suggested that autophagy role may be related to the animal model and time point. The role of autophagy during TBI maybe also dependent on the time point after TBI, which is interesting to further study in future.

## 5 CONCLUSION

In our current study, we have confirmed that Dl‐NBP has a neuroprotective effect on BBB disorders and neuron apoptosis, and subsequently contributes to motor function recovery. In addition, our data provide evidence that Dl‐NBP down‐regulates TBI/OGD‐induced autophagy and reverses sequential AJ and TJ loss and apoptosis (Figure S1C). However, the molecular mechanism underlying Dl‐NBP inhibiting autophagy remains unclear. In our next study, we will use transgenic mice, such as beclin1^−/−^ or Atg5^−/−^ mice, to deeply reveal the regulatory mechanism underlying it. Meanwhile, the effects of Dl‐NBP on mitochondrial function, inflammation and endoplasmic reticulum stress after TBI are also worthy of further investigation.

## CONFLICT OF INTEREST

The authors confirm that there are no conflicts of interest.

## AUTHOR CONTRIBUTIONS

DC, HZ and YW conceived and designed the experiments. FW and KX performed the experiments, analysed data and wrote the paper. CT, MZ, LX, RZ, LL, ZC and JX provided assistance with experiments. All authors discussed the results and approved the final manuscript.

## Data Availability

The data were used to support the findings of this study are available from the corresponding author upon request.
