# Supplementary material for: Dl‐3n‐butylphthalide improves traumatic brain injury recovery via inhibiting autophagy‐induced blood‐brain barrier disruption and cell apoptosis
Source: J Cell Mol Med. 2019 Dec 16;24(2):1220–32. doi: 10.1111/jcmm.14691 (PMC6991645; doi:10.1111/jcmm.14691)
Supplement: Supplementary file 1 [file JCMM-24-1220-s001.doc]

**Supplementary Figure1**


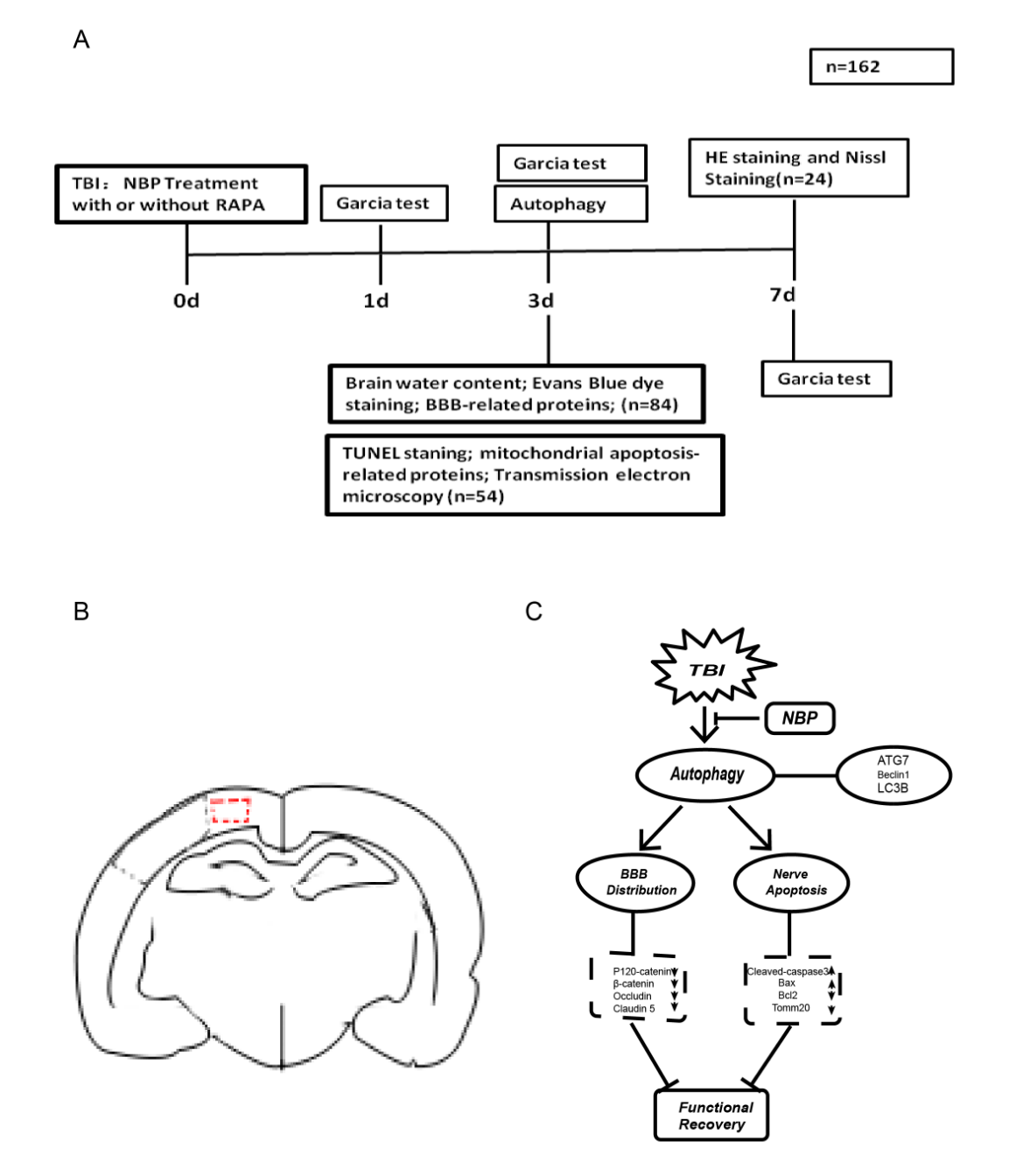


Fig. S1 The graphical time-line and schematic of current study.（A）A graphical time-line of the study.（B）The region of interest（ROI）(red) of all the immunofluorescence staining in the cortex.（C）A schematic showing the cellular molecule mechanism during Dl-NBP treatment for TBI. Dl-NBP treatment blocked autophagy-induced BBB distribution and nerve apoptosis, and thus promoted motor functional recovery after TBI in mice.

**SupplementaryFigure2**
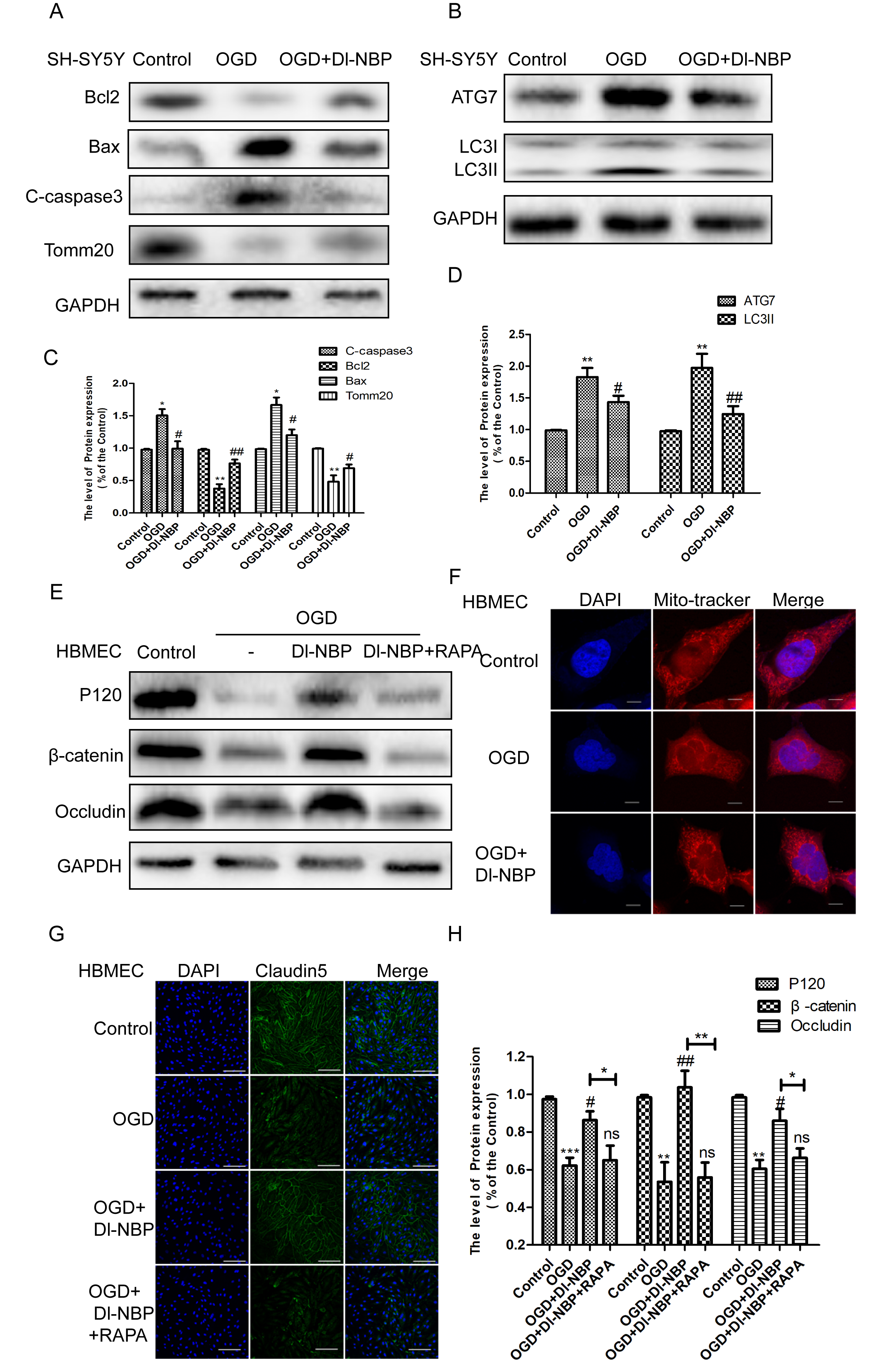


Fig. S2 Dl-NBP treatment protects OGD-treated SH-SY5Y/HBMEC cells by inhibiting autophagy in vitro. SH-SY5Y/HBMECs were treated with OGD or treated with NBP(10μM) or RAPA(100nM). (A)Representative western blots of the expression of apoptosis-related proteins and Tomm20 in SH-SY5Y cells. (B)Representative western blots of the expression of autophagy-related proteins in the SH-SY5Y cells of each group. (C,D)Quantification of the western blot data from (A,B). (E)Representative western blots of junction protein expression in HBMEC cells. (F)Mitochondrial morphologies of HBMECs exposed to either NBP(10µM) for 6h and reoxygenation for 12h or control conditions (scale bar=50μm). (G)Immunoﬂuorescence staining of claudin-5(green) and DAPI-labelled nuclei (blue) in HBMEC cells. (H)Quantification of the western blot data from (G). All data represent the mean value±SEM, and n=4 in all t-tests. **P<0.01 and ***P<0.001 vs. the control group; #P<0.05 and ##P<0.01 vs. the OGD groups; ns vs. the OGD groups; **P<0.05 and **P<0.01vs. the indicated group.
